# Disruption of Claudin-1 Expression by miRNA-182 Alters the Susceptibility to Viral Infectivity in HCV Cell Models

**DOI:** 10.3389/fgene.2018.00093

**Published:** 2018-03-20

**Authors:** Sarah E. Riad, Dalia S. Elhelw, Heba Shawer, Nada El-Ekiaby, Ayman Salah, Abdelrahman Zekri, Gamal Esmat, Asma Amleh, Ahmed I. Abdelaziz

**Affiliations:** ^1^Pharmacology and Toxicology Department, German University in Cairo, New Cairo, Egypt; ^2^Pharmaceutical Chemistry Department, German University in Cairo, New Cairo, Egypt; ^3^Biology Department, School of Science and Engineering, American University in Cairo, New Cairo, Egypt; ^4^School of Medicine, NewGiza University, Cairo, Egypt; ^5^Department of Surgery, Cairo University, Cairo, Egypt; ^6^Virology and Immunology Unit, Cancer Biology Department, National Cancer Institute, Cairo University, Cairo, Egypt; ^7^Department of Endemic Medicine and Hepatology, Cairo University, Cairo, Egypt

**Keywords:** hepatitis C virus, microRNA, entry inhibitors, Claudin-1, infection

## Abstract

HCV entry involves a complex interplay between viral and host molecules. During post-binding interactions, the viral E2 complexes with CD81 receptor for delivery to the tight junction proteins CLDN1 and OCLN, which aid in viral internalization. Targeting HCV entry receptors represents an appealing approach to inhibit viral infectivity. This study aimed at investigating the impact of targeting CLDN1 by microRNAs on HCV infectivity. miR-155 was previously shown to target the 3′UTR of CLDN1 mRNA. Therefore, miR-155 was used as a control in this study. *In-silico* analysis and luciferase reporter assay were utilized to identify potential targeting miRNAs. The impact of the identified miRNAs on CLDN1 mRNA and protein expression was examined by qRT-PCR, indirect immunofluorescence and western blotting, respectively. The role of the selected miRNAs on HCV infectivity was assessed by measuring the viral load following the ectopic expression of the selected miRNAs. miR-182 was identified *in-silico* and by experimental validation to target CLDN1. Both miR-155 and miR-182 inhibited CLDN1 mRNA and protein expression in infected Huh7 cells. Ectopic expression of miR-155 increased, while miR-182 reduced the viral load. In conclusion, despite repressing CLDN1, the impact of miR-155 and miR-182 on HCV infectivity is contradictory. Ectopic miR-182 expression is suggested as an upstream regulator of the entry factor CLDN1, harnessing HCV infection.

## Introduction

Hepatitis C Virus (HCV, order *unassigned*, family *Flaviviridae*, genus *Hepacivirus*, species *Hepatitis C virus*) entry is a multistep process requiring a coordinated interaction between viral and host factors. The virus infects the cell in the form of lipoviral particles (LVP) after association with very low-density lipoproteins (VLDL) and low-density lipoproteins (LDL) (Nielsen et al., 2006). LVPs initially interact with non-specific entry factors such as glycosaminoglycan (GAG) and LDL receptor (LDL-R) (Monazahian et al., 1999; Barth et al., 2003). This is followed by interaction with specific entry factors including the scavenger receptor B1 (SRB1), which in turn facilitates interaction of viral envelope proteins with CD81 receptor (Brazzoli et al., 2008; Dao Thi et al., 2012). Upon interaction with HCV envelope protein 2 (E2), CD81 activates Rho GTPase family members to promote actin remodeling, thus allowing the delivery of the E2-CD81 complex to come into contact with claudin-1 (CLDN1) (Brazzoli et al., 2008). CD81-CLDN1 complex then interacts with occluding (OCLN) protein. The virion translocates to the tight junctions in order to be endocytosed. Junction proteins, OCLN and CLDN1, contribute to this process (Liu et al., 2009). However, the exact mechanism by which CLDN1 mediates HCV entry is still debatable, where some studies provided evidence for a direct interaction between the Extracellular Loop 1 (EL1) of CLDN1 and HCV E1E2 complexes (Douam et al., 2014; Hopcraft and Evans, 2015), while other studies reported an indirect interaction of CLDN1 with CD81 regulating its binding to viral E2 (Brazzoli et al., 2008; Harris et al., 2008; Yang et al., 2008; Davis et al., 2012; Farquhar et al., 2012; Bonander et al., 2013). In addition to its role in HCV entry, CLDN1 was also shown to be essential for HCV spread between hepatocytes via cell-cell transmission (Timpe et al., 2008).

Hence, claudin-1 (CLDN1) is an essential co-receptor for HCV entry. It is a tight junctional protein, that is highly expressed in the liver (Evans et al., 2007), localized both at the apical-canalicular tight junction region and at basolateral-sinusoidal hepatocyte surfaces (Reynolds et al., 2008). The highly conserved EL1 was found to be recruited in viral entry (Evans et al., 2007). Several studies provide proofs for the critical role of CLDN1 during the HCV entry process, where its expression in CLDN1 defective mutants of HCV resistant Huh7 cell lines restored permissiveness to HCV (Fukasawa, 2014). Interestingly, it was found to be the first factor to confer susceptibility to HCV when ectopically expressed in non-hepatic cells and non-permissive cell lines (Evans et al., 2007).

Intriguingly, monoclonal antibodies against CLDN1 were shown to inhibit HCV entry, cell-cell-transmission and virus-induced signaling events in absence of detectable toxicity (Mailly et al., 2015; Yamashita et al., 2015), and they were also effective in hindering infection by all major HCV genotypes and quasispecies (Fofana et al., 2010). Moreover, when combined with direct acting antivirals or host targeting agents, a synergistic inhibition of HCV infection was attained (Xiao et al., 2015). Several compounds were reported to target CLDN1 inhibiting viral infection, either by inhibiting its co-localization with CD81, such as EWI-2wint (Potel et al., 2013); reducing its expression, such as PKA antagonist and aspirin (Mee et al., 2009; Yin and Zhang, 2016); or modifying its cellular localization, such as sorafenib (Descamps et al., 2015).

However, regulation of hepatic CLDN1 expression by microRNAs was not clearly investigated in HCV infection, but it was just investigated in liver cancer, showing that both miR-198 and miR-199a enhanced CLDN1 expression (Elfimova et al., 2013; Suzuki et al., 2014).

Previous studies showed contradicting impacts of miR-155 on CLDN1 expression in different disease states, where on the one hand, overexpression of miR-155 upregulated CLDN1 expression in colorectal cancer (Zhang et al., 2013), while on the other hand, repressed expression of miR-155 was correlated with CLDN1 overexpression, in ovarian cancer-initiating cells. However, in the latter study miR-155 was proven to target the 3′UTR of CLDN1 by the luciferase reporter assay (Qin et al., 2013). Nevertheless, during HCV-infection, miR-155 CLDN1 interaction is still elusive.

In this study, miR-182 was predicted by bioinformatics to target CLDN1. miR-182 was previously shown to have an impact on HCV replication, where it was shown that viral replication within Huh7 cells decreased upon co-culture with NK cells, in which miR-182 was overexpressed by transfection with miR-182 mimics. However, ectopic expression of miR-182 in infected Huh7 cells, increased viral replication of genotypes 2 and 4 suggesting “contradicting roles” of miR-182 on viral replication. Moreover, miR-182 was predicted to have binding sites on the viral genome (El Sobky et al., 2016). miR-182 was also found to be implicated in other viral infections, where it was found to be overexpressed in fibroblasts infected with human cytomegalovirus (CMV) (Stark et al., 2012) and its suppression inhibited HIV-1 replication (Chen et al., 2013). However, its regulatory impact on CLDN1 was not previously investigated.

Thus, the main aim of this study was to investigate the regulation of CLDN1 by ectopic expression of both miR-155, as a control, and miR-182, as a novel miRNA, and their impact on HCV infectivity.

## Patients and methods

### Study subjects

A total of 17 fine needle biopsies were obtained from liver tissues of HCV-infected patients. For all the patients, HCV infection was diagnosed by the presence of anti-HCV antibodies and HCV RNA in the serum. All patients were non-cirrhotic, HBV negative and treatment-naïve. Healthy liver biopsies were obtained from ten liver tissue samples of healthy donors before transplant into the recipients during liver transplantation. All the experiments were performed in compliance with the guidelines of the Institutional Review Board of Kasr El Aini Medical School in Cairo University (Cairo, Egypt) and in accordance with the Declaration of Helsinki. Written informed consent was obtained from all patients included in the study.

### Bioinformatics analysis

In order to identify candidate miRNAs, that target CLDN1 via 3′UTR interactions, several computational algorithms were utilized, including miRanda, TargetScan, mirDIP, and miRwalk. Selection criteria for miRNAs included in this study were lowest hybridization energy, highest binding scores and the number of algorithms that agree upon them.

### Huh7 cell culture

Huh7 cell lines were grown at 37°C in a 5% carbon atmosphere in Dulbecco's modified Eagle's medium (DMEM; Invitrogen; Carlsbad, CA, USA) supplemented with 4.5 g L^−1^ Glucose, L-Glutamine, and 10% Fetal Bovine Serum (FBS; Invitrogen; USA), 1% streptomycin/penicillin (Invitrogen; USA) and Mycozap (Lonza; Basel, Switzerland).

### Luciferase reporter assay

To confirm binding of miR-182 to CLDN1 3′UTR, firefly luciferase reporter vector was used (pmirGLO) (Promega; Madison, WI, USA). The complementary target site for miR-182 on CLDN1 3′UTR, predicted using *in silico* analysis, was flanked by sticky ended 5′SacI and 3′XbaI restriction sites to form the wild-type (WT) insert, or the binding site was deleted to form the mutant type insert (MT). pmirGLO was double digested using XbaI and SacI (Thermo Scientific; Waltham, MA, USA) restriction enzymes. This was followed by ligation of either WT or MT inserts using T4 Ligase (Takara Shuzo Co. Ltd., Kyoto, Japan). Forward (F) and reverse (R) primers' sequences were designed as follows: for the WT target site

F 5′CATCTTTCTACCTCTTTTTTCTATCTGCCAAATTGAGATAAT3′
R 5′CTAGATTATCTCAATTTGGCAGATAGAAAAAAGAGGTAGAAAGATGAGCT3′
and for the MT target site
F 5′CATCTTTCTACCTCTTTTTTCTATCATTGAGATAAT3′
R 5′CTAGATTATCTCAATGATAGAAAAAAGAGGTAGAAAGATGAGCT3′.

To ensure insert ligation, the empty, as well as wild and mutant ligated pmirGLO constructs were subjected to XhoI (Thermo Scientific; USA) digestion. Since the XhoI restriction site is located between SacI and XbaI restriction sites, only the empty pmiRGlo vector was digested with XhoI enzyme, while the ligated pmirGLO constructs harboring the WT/MT inserts were not digested, confirming insert ligation. Huh7 cells were transfected with either empty pmirGLO vector or pmirGLO constructs harboring WT or MT inserts using SuperFect transfection reagent (Qiagen; Hilden, Germany). After 24 h, cells were either co-transfected with miR-182 mimics using Hiperfect transfection reagent (Qiagen, Germany) or kept untransfected. Luciferase activity was measured 48 h post-transfection using the Luciferase reporter assay kit (Biovision Technologies; California, USA).

### HCV constructs

The pJFH plasmid harboring the genotype 2a genome (kindly provided by Professor T. Wakita) and the intergenotypic recombinant pED435′UTR-NS2/JFH1T827A, T977S harboring the genotype 4a genome (kindly provided by Professor J. Bukh) were linearized using the XbaI restriction enzyme (ThermoScientific; USA) and purified using the phenol-chloroform technique. The full length viral RNA was *in vitro* transcribed using the T7 polymerase kit (MEGAscript, Ambion, Life Technologies; Carlsbad, CA, USA) according to the manufacturer's instructions.

### Preparation of HCVcc

The transcribed viral genome was delivered into Huh7 cells by transfection (SuperFect; Qiagen, Germany) according to the manufacturer's instructions. Supernatants harboring the released HCV particles were collected 72 h post-transfection, filtered through 0.45 μm pore size filters and stored at −80°C for further use.

### RNA extraction

Total RNA was extracted from liver tissues using mirVana Isolation Kit (Ambion; Austin, TX, USA) and from Huh7 cells using the Biozol extraction reagent (Bioer Technology Co., Ltd., Hangzhou, China) according to manufacturer's instructions.

### Reverse transcription and qRT-PCR

Total RNA was reverse transcribed into single-stranded complementary DNA (cDNA) using the high-capacity cDNA reverse transcription kit (Applied Biosystems; Foster City, CA, USA) following manufacturer's instructions. miR-155-5p, miR-182-5p, as well as the reference miRNA, RNU6B, were reverse transcribed using TaqMan MicroRNA Reverse Transcription Kit and Taqman specific stem-loop primers (Applied Biosystems; USA) following manufacturer's instructions. Real-time PCR was performed using qPCR with Taqman probes (Applied Biosystems; USA) and StepOne PCR (Applied Biosystems; USA). miRNA expression was normalized to RNU6B and mRNA expression was normalized to Beta-2-Microglobulin (B2M).

### Delivery of oligonucleotides into Huh7 cells

5 × 10^4^ cells seeded for 24 h in a 96-well plate were either transfected with siRNA against CLDN1, miR-155 mimic/antagomirs or miR-182 mimics/antagomirs (Qiagen; Germany). Transfection was performed using Hiperfect transfection reagent (Qiagen; Germany) according to manufacturer's instructions. CLDN1 expression was evaluated on the mRNA level 48 h and on the protein level 72 h post-transfection.

### Indirect immunofluorescence

72 h post-transfection cells were fixed using 4% paraformaldehyde. Then, cells were permeabilized using 0.05% Tween 20. After blocking with Bovine Serum Albumin, CLDN1 specific primary antibody (Santa Cruz Biotechnology; Dallas, TX, USA, sc-166338, A-9, mouse monoclonal IgG_2b_,) was added. Cells were then incubated with TexasRed-conjugated secondary antibody (Santa Cruz Biotechnology USA, Goat anti-mouse IgG) and mounted with DAPI mounting medium. For quantifying the fluorescence of immunolabeled CLDN1 in each well, Perkin Elmer Wallac 1420 VICTOR2™ microplate reader (GMI, Inc.; Ramsey, MN, USA) was used.

### Western blot analysis

Cells were lysed in ice-cold Laemmli lysis buffer with freshly added 1xHalt Protease Inhibitor Cocktail (ThermoScientific, USA) and protein concentration was determined using BCA Protein Assay Kit (Pierce Biotechnology; Waltham, MA, USA) according to the manufacturer's instructions. Equal concentrations (40 μg) were separated by 12% SDS-PAGE gels and then blotted onto polyvinylidene difluoride membrane. The membrane was blocked using 5% nonfat dry milk and incubated overnight with the anti-CLDN1 primary antibody (Santa Cruz Biotechnology; USA, 1:100) at 4°C. β-tubulin (Sigma; Missouri, USA, T7816; mouse monoclonal IgG1; 1:20,000) was used as loading control. The membrane was then incubated with the anti-mouse alkaline phosphatase-conjugated secondary antibody (KPL, Maryland, USA) after thorough washing. The protein bands were detected using Phosphatase Chemiluminescent Substrate.

### Huh7 cell infection and viral RNA extraction

To assess the impact of miR-155 and miR-182 on viral infectivity, 2.5 × 10^5^ cells were seeded in a 24-well plate, followed by transfection with the respective mimics. 48 h post-transfection Huh7 were infected with equal amounts of JFH1 or ED43/JFH1 HCVcc (200 μl) for 24 h. Viral RNA was extracted using the Invisorb Spin virus RNA mini kit (Invitek; Berlin, Germany) according to manufacturer's protocol. RNA was stored at −80°C until further use.

### HCV RNA quantification

Viral RNA was quantified using the HCV Real-Time PCR Detection Kit (BIOER, China) using standards of known quantities according to manufacturer's instructions. Viral load was calculated as percentage of the untransfected HCV-infected cells.

### Statistical analysis

Data was analyzed using Prism 5 software, version 5.00 (GraphPad Software; San Diego, CA, USA). *p* < 0.05 were considered statistically significant (^***^p < 0.001, ^**^p < 0.01, ^*^p < 0.05 and ns, statistically not significant). Unpaired *t*-test was used to draw comparisons between samples. Relative Quantitation (RQ) is used for expression of the qRT-PCR results, calculated using the ΔΔCT method [RQ = 2^−ΔΔCT^]. Results are expressed as the mean ± standard error of the mean (SEM).

## Results

### Bioinformatics analysis and luciferase reporter assay

**Figure 1:**
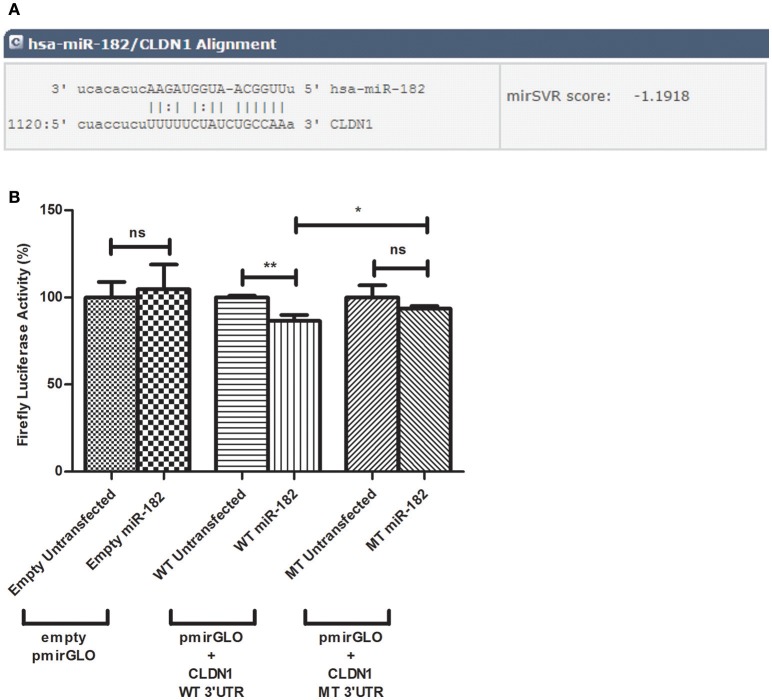
Predicted alignment and luciferase reporter assay. **(A)** miR-182 targets CLDN1 3′UTR at position 1120 with a mirSVR score of −1.918 as predicted by microRNA.org. **(B)** A decline in luciferase activity is evident in Huh7 cells co-transfected with miR-182 mimic and pmirGLO vector harboring the wild type (WT) insert (*N* = 6) (*p* = 0.0035^**^), compared to WT untransfected cells (*N* = 6). However, miR-182 mimic does not alter luciferase activity in either empty pmirGLO (*N* = 9) or pmirGLO vector harboring the mutant type (MT) insert (*N* = 9), each compared to un-mimicked cells (*N* = 9). Comparing firefly luciferase activity in miR-182 transfected cells containing wild-type to those containing mutant type 3′UTR, a moderate but significant reduction is also evident (*p*-value of 0.0442^*^). ^**^p < 0.01, ^*^p < 0.05 and ns, statistically not significant.

Potential microRNAs that target the 3′UTR of CLDN1 were identified using the *in silico* analysis tools, miRanda, TargetScan, mirDIP, and miRwalk, which agreed upon the prediction of miR-182 to target the 3′UTR of CLDN1 with a high binding score (Figure 1A). The selection of miR-182 was also supported by its reported role in immunoregulation and viral infections.

To investigate potential binding of miR-182 to CLDN1 at the predicted site within the 3′UTR of CLDN1, the luciferase reporter assay was used. After transfection with the different pmirGLO constructs, the percentage decline in firefly luciferase activity was assessed in miR-182-mimicked cells normalized to un-mimicked cells. miR-182 mimics did not reduce luciferase activity in cells transfected with an empty (*N* = 9) or mutant insert containing pmirGLO (*N* = 9) compared to unmimicked empty (*N* = 9) or mutant insert containing pmirGLO (*N* = 9), respectively. Cells co-transfected with miR-182 mimics and wild-type pmirGLO reduced the luciferase activity. Although the reduction was only moderate, it was statistically significant (*p* = 0.0035^**^) (*N* = 6) compared to unmimicked cells (*N* = 6). A statistically significant decrease in firefly luciferase activity was also evident in miR-182 transfected cells containing WT compared to MT 3′UTR (*p* = 0.0442^*^) (Figure 1B).

### Expression profile of CLDN1 and miR-182 in infected liver biopsies and Huh7 cells

Relative expression of CLDN1 normalized to the housekeeping gene B2M and miR-182 normalized to RNU6B was assessed in liver tissue samples from HCV-infected patients and compared to liver tissues from healthy controls. No significant difference was observed in CLDN1 mRNA expression in HCV patients compared (1.085 ± 0.1792, *N* = 17) to healthy controls (1.281 ± 0.2872, *N* = 10). miR-182 (0.4226 ± 0.1225, *N* = 17, *p* = 0.0211^*^) was found to be downregulated in HCV-infected patients compared to healthy controls (2.498 ± 1.095, *N* = 10).

**Figure 2:**
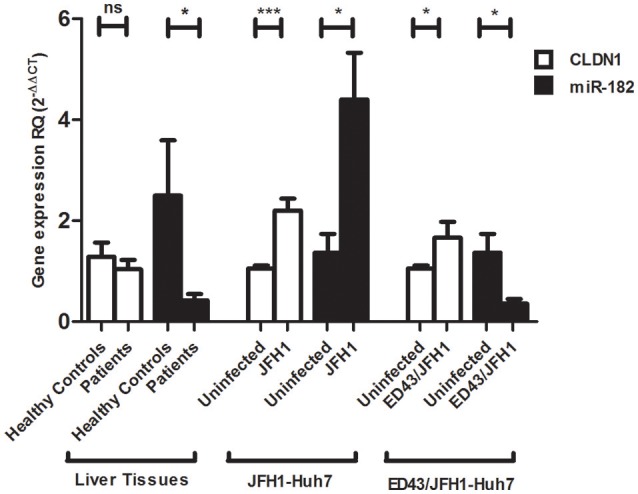
Expression profile of CLDN1 and miR-182 in infected liver biopsies and Huh7 cells. No difference in CLDN1 mRNA expression is observed in liver biopsies of HCV patients (*N* = 17) compared to healthy controls (*N* = 10), while miR-182 (*N* = 17, *p* = 0.0211*) is downregulated in HCV-infected patients compared to healthy controls (*N* = 10). Higher expression of CLDN1 mRNA is observed in JFH1- (*p* < 0.0001***) and ED43/JFH1- (*p* = 0.0429*) infected compared to uninfected Huh7 cell lines. miR-182 shows higher expression levels in JFH1-infected Huh7 cells (*p* = 0.0156*) and reduced expression in ED43/JFH1-infected Huh7 cells (*p* = 0.0104*) compared to naïve Huh7 cells. Relative Quantitation (RQ) is used for expression of the results. ***p < 0.001, *p < 0.05 and ns, statistically not significant.

In Huh7 cells, relative expression of CLDN1 and miR-182 was investigated in uninfected cells, as well as JFH1- and ED43/JFH1-infected cell models. JFH1- (2.204 ± 0.2312, *p* < 0.0001^***^) and ED43/JFH1- (1.662 ± 0.3150, *p* = 0.0429^*^) infected cells showed significantly higher expression of CLDN1 compared to uninfected cells (1.054 ± 0.06049 and 1.054 ± 0.06049, respectively). miR-182 was found to be induced (4.402 ± 0.9250, *p* = 0.0156^*^) upon JFH1 infection, but significantly repressed (1.370 ± 0.3699, *p* = 0.0104^*^) upon ED43/JFH1 infection, when compared to naïve Huh7 cells (1.370 ± 0.3699 and 0.3580 ± 0.09183, respectively) (Figure 2).

### Impact of miR-155 and miR-182 on CLDN1 expression in infected Huh7 cell models

**Figure 3:**
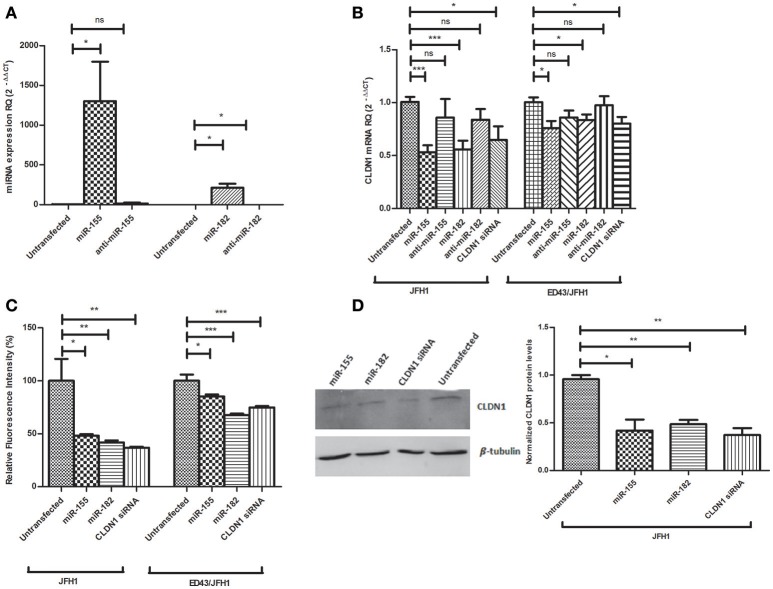
Impact of miR-155 and miR-182 on CLDN1 expression in infected Huh7 cell models. **(A)** A significant upregulation in the expression level of miR-155 (*p* = 0.0307*) and miR-182 (*p* = 0.0127*) is observed upon mimicking of Huh7 cells as compared to untransfected cells. A non-significant reduction in miRNA expression is observed with anti-miR-155 and a significant reduction is evident with anti-miR-182 (*p* = 0.0210*) **(B)** miR-155 (*p* = 0.0002***) and miR-182 mmics (*p* = 0.0009***) decrease CLDN1 mRNA expression in JFH1-infected cells. Also in ED43/JFH1-infected cells, miR-155 (*p* = 0.0179*) and miR-182 (*p* = 0.0395*) mimics decrease CLDN1 mRNA expression. Antagomirs against both miRNAs restored CLDN1 mRNA expression during both JFH1 and ED43/JFH1 infections to a level not significantly different than untransfected cells. Knocking down CLDN1 using siRNAs as a positive control decreases the expression of CLDN1 mRNA in both JFH1- (*p* = 0.0203*) and ED43/JFH1- (*p* = 0.0339*) infected cells. Relative Quantitation (RQ) is used for expression of the results. **(C)** Percentage fluorescence intensity of the immunolabeled CLDN1 protein is plotted. miR-155 (*p* = 0.0183*) and miR-182 mmics (*p* = 0.0091**) decrease CLDN1 protein levels in JFH1-infected cells. miR-155 (*p* = 0.0119*) and miR-182 (*p* < 0.0001***) mimics decrease CLDN1 protein levels in ED43/JFH1-infected cells. Knocking down CLDN1 using siRNAs decreases the expression CLDN1 protein in both JFH1 (0.0049**) and ED43/JFH1 (*p* < 0.0001***)-infected cells. **(D)** Protein levels of CLDN1 in infected Huh7 cells transfected with miR-155, miR-182 mimics or CLDN1 siRNA is assessed by western blotting, and the density of the bands is measured and plotted on a graph. Levels of CLDN1 protein (Mwt: 22.7 kDa) are normalized to levels of β-tubulin (Mwt: 55 kDa) in the same wells. Cells transfected with miR-155 (*p* = 0.0121*) and miR-182 mimics (*p* = 0.0016**) show significantly lower levels of CLDN1 protein as compared to untransfected cells. siRNAs against CLDN1 efficiently decreased its protein levels (*p* = 0.0022**) compared to untransfected cells. ***p < 0.001, **p < 0.01, *p < 0.05 and ns, statistically not significant.

To confirm successful delivery of the oligos into Huh7 cells, the expression of both miRNAs, normalized to RNU6B, was assessed 48 h post-transfection with the mimics or antagomirs. The expression of miR-155 (1,302 ± 496.4, *p* = 0.0307^*^) and miR-182 (213.3 ± 49.46, *p* = 0.0127^*^) was enhanced in mimicked Huh7 cells compared to untransfected cells (2.338 ± 1.617 and 213.3 ± 49.46, respectively) by 557 and 201 folds, respectively. Antagomirs reduced the expression of the miRNAs, however without statistical significance in case of anti-miR-155 and with significance for anti-miR-182 (*p* = 0.0210^*^) (Figure 3A).

After confirmation of transfection, the impact of miR-155 and miR-182 on CLDN1 mRNA was assessed by transfection of JFH1- or ED43/JFH1-infected Huh7 cells with miR-155/miR-182 mimics or antagomirs, or CLDN1 siRNA as a positive control. In JFH1-infected cells, both miR-155 (0.5319 ± 0.06531, *p* = 0.0002^***^) and miR-182 mimics (0.5572 ± 0.08326, *p* = 0.0009^***^) were found to significantly reduce CLDN1 mRNA expression compared to untransfected cells (1.006 ± 0.04874), while anti-miR-155 (0.8599 ± 0.1740) and anti-miR-182 (0.8379 ± 0.1012) restored the expression of CLDN1 mRNA to a level not significantly different than the untransfected cells (1.006 ± 0.04874). Similarly, miR-155 (0.7600 ± 0.06671, *p* = 0.0179^*^) and miR-182 (0.8372 ± 0.05054, *p* = 0.0395^*^) suppressed CLDN1 mRNA in ED43/JFH1-infected cells compared to untransfected cells (1.004 ± 0.04537 and 1.008 ± 0.04084, respectively), while anti-miR-155 (0.8607 ± 0.06483) and anti-miR-182 (0.9757 ± 0.08543) also restored CLDN1 expression to a level comparable to untransfected cells (1.006 ± 0.04874). siRNA against CLDN1 efficiently knocked down its expression in both JFH1- (0.6478 ± 0.1280, *p* = 0.0203^*^) and ED43/JFH1- (0.8020 ± 0.06300, *p* = 0.0339^*^) infected Huh7 cells compared to untransfected JFH1- (1.006 ± 0.04874) and ED43/JFH1-infected cells (1.004 ± 0.04537), respectively (Figure 3B).

To investigate the impact of miR-155 and miR-182 on CLDN1 protein, fluorescence of the immunolabeled CLDN1, was measured in JFH1-, as well as ED43/JFH1-HCV-infected Huh7 cells, transfected with miR-155/miR-182 or CLDN1 siRNA and quantified by the microplate reader. TexRed fluorescence intensity was normalized to the DAPI fluorescence. In JFH1-infected Huh7 cells, fluorometric quantification revealed that both miR-155 (48.16 ± 1.572, *p* = 0.0183^*^) and miR-182 mimics (41.96 ± 1.836, *p* = 0.0091^**^) significantly downregulate CLDN1 protein compared to untransfected cells (100.0 ± 20.63). Likewise, miR-155 (85.14 ± 2.088, *p* = 0.0119^*^) and miR-182 (67.71 ± 1.392, *p* < 0.0001^***^) reduced CLDN1 protein levels in ED43/JFH1-infected Huh7 cells compared to untransfected cells (100.0 ± 5.931). siRNA against CLDN1 efficiently knocked down its expression in both JFH1- (36.98 ± 0.8557, *p* = 0.0049^**^) and ED43/JFH1- (74.78 ± 1.531, *p* < 0.0001^***^) infected Huh7 cells compared to untransfected JFH1- (100.0 ± 20.63) and ED43/JFH1-infected cells (100.0 ± 5.931), respectively (Figure 3C).

To further confirm the impact of miR-155 and miR-182 on CLDN1 protein, western blot analysis was performed. β-tubulin was used as a loading control. Cells mimicked with miR-155 (0.4200 ± 0.1153, *p* = 0.0121^*^), as well as miR-182 (0.4867 ± 0.04372, *p* = 0.0016^**^), showed a significant downregulation of CLDN1 protein compared to untransfected cells (0.9567 ± 0.04333). CLDN1 siRNA (0.3733 ± 0.07172, *p* = 0.0022^**^), which was used as a positive control, also reduced CLDN1 protein levels compared to untransfected cells (0.935 ± 0.065) (Figure 3D). Original gel images are provided as Supplementary Material (Supplementary Image 1).

### Impact of miR-155 and miR-182 on CLDN1 expression in Naïve Huh7 cells

**Figure 4:**
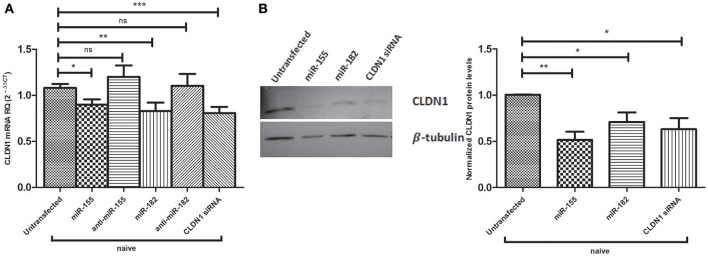
Impact of miR-155 and miR-182 on CLDN1 expression in naïve Huh7 cells. **(A)** Both miR-155 (*p* = 0.0173*) and miR-182 mimics (*p* = 0.0071**) significantly reduce CLDN1 mRNA expression compared to untransfected cells). Antagomirs against miR-155 and miR-182 restore CLDN1 mRNA expression to a level not significantly different than the untransfected cells. Knocking down CLDN1 using siRNAs suppresses CLDN1 mRNA levels in transfected Huh7 cells (*p* = 0.0007***) compared to untransfected cells. Relative Quantitation (RQ) is used for expression of the results. **(B)** Western blot analysis shows that cells transfected with miR-155 (*p* = 0.0014**) and miR-182 mimics (*p* = 0.0308*) show significantly lower levels of CLDN1 protein as compared to untransfected cells. CLDN1 siRNA (*p* = 0.0195*) also reduce CLDN1 protein levels compared to untransfected cells. ***p < 0.001, **p < 0.01, *p < 0.05 and ns, statistically not significant.

The impact of miR-155 and miR-182 on CLDN1 expression was also assessed in naïve Huh7 cells, both on the mRNA and protein levels (Figure 4). Both miR-155 (0.8989 ± 0.05918, *p* = 0.0173^*^) and miR-182 mimics (0.8289 ± 0.09232, *p* = 0.0071^**^) were found to significantly reduce CLDN1 mRNA expression compared to untransfected cells (1.082 ± 0.042), while anti-miR-155 (1.201 ± 0.1247) and anti-miR-182 (1.105 ± 0.1272) restored the expression of CLDN1 mRNA to a level not significantly different than the untransfected cells (1.082 ± 0.042) siRNA against CLDN1 suppressed its expression in transfected Huh7 cells (0.8083 ± 0.06737, *p* = 0.0007^***^) compared to untransfected cells (1.082 ± 0.042) (Figure 4A). Western blot analysis showed that cells mimicked with miR-155 (0.5175 ± 0.08740, *p* = 0.0014^**^), as well as miR-182 (0.7100 ± 0.1050, *p* = 0.0308^*^), exhibit a significant downregulation of CLDN1 protein compared to untransfected cells (1.005 ± 0.002887). CLDN1 siRNA (0.6325 ± 0.1177, *p* = 0.0195^*^) also reduced CLDN1 protein levels compared to untransfected cells (1.005 ± 0.002887) (Figure 4B).

### Impact of miR-155 and miR-182 on HCV infectivity

**Figure 5:**
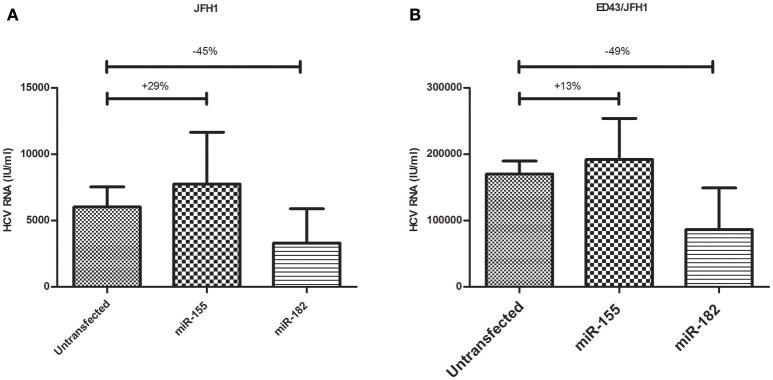
Impact of miR-155 and miR-182 on HCV infectivity. **(A)** miR-155 mimicked Huh7 cells (*N* = 3) show higher levels of HCV RNA, while miR-182 mimicked cells (*N* = 3) show reduced HCV RNA compared to untransfected cells (*N* = 4) both infected with HCVcc derived from JFH1. **(B)** Upon infection with HCVcc derived from ED43/JFH1, cells mimicked with miR-155 (*N* = 3) showed increased levels of HCV RNA, while cells mimicked with miR-182 (*N* = 3) showed reduced levels of HCV RNA compared to untransfected cells (*N* = 3) both infected with ED43/JFH1 supernatants.

Since miR-155 and miR-182 were shown to downregulate CLDN1, an important HCV entry factor, it was important to assess their impact on HCV infectivity. Naïve Huh7 cells were either transfected with miR-155 or miR-182 mimics or kept untransfected. 48 h post-transfection, cells were incubated for 24 h with HCVcc derived from either JFH1 or ED43/JFH1. This was followed by viral RNA extraction and qRT-PCR. Interestingly, miR-182-mimicked Huh7 cells were found to be less vulnerable to HCV infection by HCVcc either derived from JFH1 or ED43/JFH1 compared to untransfected cells, reducing HCV infectivity by 45 and 49%, respectively. However, miR-155 mimicked cells showed an increase in the viral load upon JFH1, as well as ED43/JFH1 infection by 29 and 13%, respectively when compared to untransfected cells (Figure 5).

## Discussion

HCV entry into hepatocytes represents a pivotal step in the viral lifecycle essential for the initiation, spread, and maintenance of infection. CLDN1 is a surface and tight junctional protein (Reynolds et al., 2008), that has been described as the first factor to confer susceptibility to HCV when ectopically expressed in non-hepatic cells (Evans et al., 2007). Therefore, this work aimed at studying the regulation of the host entry factor CLDN1 by miRNAs in an attempt to understand its impact on HCV infectivity.

miR-155 was previously confirmed to bind to the 3′UTR of CLDN1 in non-HCV studies (Qin et al., 2013). However, its impact on the HCV lifecycle was not investigated. Taking miR-155 as a validated control in this study, bioinformatics analysis was performed, which revealed that miR-182 potentially targets the 3′UTR of CLDN1 mRNA with high binding affinity. In an attempt to assess, whether the impact of miR-182 on CLDN1 is mediated via a direct binding of the miRNA to the 3′UTR of CLDN1, a luciferase reported assay was performed with a construct harboring wild type and mutant sequences of the 3′UTR of CLDN1. A significant reduction in luciferase activity was observed in the wild type construct.

On the other hand, liver tissue expression-profiling was performed for both miR-182 and CLDN1. For CLDN1, its expression revealed no significant change in the livers of HCV-infected patients compared to healthy liver tissues. These findings simulate another study performed by Holczbauer et al., which showed no difference in CLDN1 expression in HCV-positive compared to HCV-negative liver tissues (Holczbauer et al., 2014). However, it stands in contradiction with a couple of other studies showing differential expression of CLDN1 in HCV-infected liver tissues (Nakamuta et al., 2011; Haid et al., 2014). For further confirmation, JFH1- and ED43/JFH1-infected Huh7 cell lines were assessed for CLDN1 expression and they both showed a significant upregulation in CLDN1 expression compared to naïve cells. These findings are in agreement with other studies showing increased CLDN1 expression (Reynolds et al., 2008), while they disagree with studies showing a decrease in CLDN1 in HCV-infected cell models (Liu et al., 2009). This controversial pattern of expression may be due to the overgrowth of uninfected cells with lower CLDN1 expression, overriding the death of HCV-infected cells with higher CLDN1 expression resulting in a decline in the mean CLDN1 expression with time (Padmanabhan and Dixit, 2012). On the other hand, it was stated that HCV infection does increase CLDN1 expression, but it is followed by a compensatory decline in CLDN1 expression to prevent superinfection (Liu et al., 2009). Thus, it can be concluded from present and previous data that the level of CLDN1 might be dependent on the state of HCV infection.

In a similar approach, miR-182 was also screened in liver tissues, as well as HCV cell models. miR-182 showed a reduced expression in HCV-infected liver tissues, as well as genotype 4 (ED43/JFH1)-infected cell lines and an increased expression in genotype 2 (JFH1)-infected cells. These findings are supported by another study, which reported a comparable decreased expression of miR-182 in genotype 4-infected liver tissues and cell lines (El Sobky et al., 2016). Overexpression of miR-182 in HCV-genotype 2 infection stands in concordance with the reported induced expression of miR-182 in other viral infections such as cytomegalovirus (CMV) infection (Stark et al., 2012). The unique suppression of miRNAs encountered during genotype 4-HCV infection was previously reported with miR-155, which showed increased expression in genotypes 1, 2, and 3-infected liver tissues (Zhang et al., 2012; Jiang et al., 2014), and Huh7 cell lines (Zhang et al., 2012; Riad et al., 2015), but a repressed induction in genotype 4-infected liver tissues and genotype 4-infected cell lines. This suppression was suggested to be uniquely attributed to genotype 4-HCV infection (Riad et al., 2015), mediated by viral interference with miR-155 expression at a site upstream of IFN release within the TLR-7 pathway (El-Ekiaby et al., 2012).

Ectopic expression of miR-182 led to repression of CLDN1 on both mRNA and protein levels in both JFH1- and ED43/JFH1-infected Huh7 cell models. Similarly, forcing miR-155 expression led to a reduction in CLDN1 on the mRNA and protein levels. This reduction in CLDN1 expression by miRNAs was also evidenced in other non-HCV studies, where miR-375 and miR-29a showed a marked depression in CLDN1 expression (Yoda et al., 2014; Mahati et al., 2017). Reduction in CLDN1 mRNA and protein expression was stronger compared to the reduction observed in the luciferase reporter assay. Thus, although the direct impact of miR-182 on CLDN1 might be only moderate, it seems that other mechanisms might contribute to the significant reduction in CLDN1 expression observed upon ectopic expression of miR-182 in naive, genotype 2 and genotype 4 infected cells. Therefore, we do not exclude the possibility that the impact of miR-182 on CLDN1 might be via an additive direct and indirect effect. Interestingly, miR-182 was found to have potential binding sites on Occludin co-entry factor (microRNA.org). It was also found to have some binding sites on the virus itself (VBRC; https://virology.uvic.ca/). Those interactions need to be further experimentally validated, which may contribute to an indirect impact of miR-182 (Supplementary Document 1). Further studies might be required to assess other contributing factors.

The correlation between the regulation of CLDN1 expression by microRNAs and HCV infectivity was then assessed by forcing the expression of both miRNAs, miR-155 and miR-182, in Huh7 cells followed by infection with JFH1 and ED43/JFH1 HCVcc and evaluation of the viral infectivity by quantifying intracellular viral particles.

Results showed a discrepancy in viral infectivity, where miR-155 caused an increase in the viral load, despite repressing CLDN1. This could be explained by previous literature showing a positive correlation between miR-155 expression and HCV replication (Grek et al., 2011). Thus, miR-155 may reduce HCV entry, but its subsequent induction of HCV replication might mask its entry-impeding effect.

On the other hand, miR-182 behaved in opposition to miR-155, showing a reduction in HCV infectivity upon infection with HCVcc derived from JFH1, as well as ED43/JFH1. These findings suggest miR-182 may act as a novel HCV-infection-inhibiting miRNA, which acts by targeting the HCV entry receptor, CLDN1. This work goes in similar with our group's work where a reduction in permissiveness to HCV was reported by miR-194, which was found to suppress the vulnerability to HCV infection either by JFH1 or ED43/JFH1 through targeting CD81.

These results also go in parallel with previous studies targeting CLDN1, where monoclonal antibodies against CLDN1 inhibited HCV entry and all HCV related signaling events (Mailly, Xiao et al., 2015; Yamashita et al., 2015), and were also effective in hindering infection by all major genotypes of HCV (Fofana et al., 2010).

In conclusion, this study aimed at testing the regulation of the tight junction protein, CLDN1, by miRNAs in HCV-infected cell replicons, revealing similar impact of ectopic miR-155 and miR-182 on CLDN1 repression and contradicting roles on HCV infectivity. This data highlights the role of exogenously-induced miR-182 as a suppressor of HCV infection for further investigations as a potential novel therapeutic approach.

## Data availability statement

All datasets supporting the conclusions for this study are included in the manuscript.

## Ethics statement

This study was carried out in accordance with the recommendations of guidelines of the Institutional Review Board of Kasr El Aini Medical School in Cairo University (Cairo, Egypt) with written informed consent from all subjects. All subjects gave written informed consent in accordance with the Declaration of Helsinki. The protocol was approved by the Institutional Review Board of Kasr El Aini Medical School in Cairo University (Cairo, Egypt).

## Author contributions

SR performed the experimental work, analyzed data and wrote manuscript; DE and HS contributed in the experimental work; NE-E designed experiments; AS and AZ provided samples and performed experiments; AA supervised Western Blot experiments; GE co-supervised the study; AIA conceived and supervised the study.

### Conflict of interest statement

The authors declare that the research was conducted in the absence of any commercial or financial relationships that could be construed as a potential conflict of interest.

## Supplementary material

Supplementary Image 1 Original full-length western blots used for Figure 3D. **(A)** Original western blot of Huh7 cell lysates treated with anti-CLDN1 antibodies. **(B)** Western blot corresponding to membrane treated with anti-β-tubulin antibodies.
