# Supplementary figures and images for: Disruption of Claudin-1 Expression by miRNA-182 Alters the Susceptibility to Viral Infectivity in HCV Cell Models
Source: Front Genet. 2018 Mar 20;9:93. doi: 10.3389/fgene.2018.00093 (PMC5869927; doi:10.3389/fgene.2018.00093)

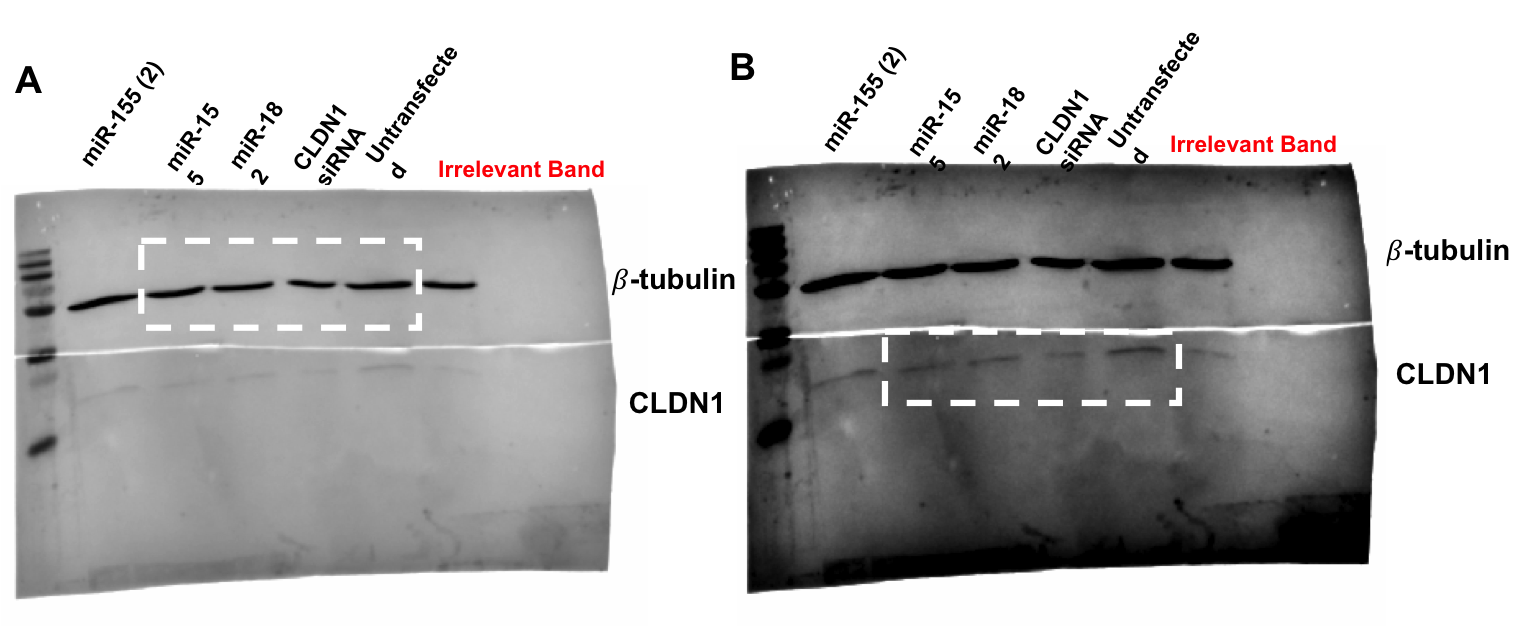

Supplement: Supplementary Image 1 — Original full-length western blots used for Figure 3D. (A) Original western blot of Huh7 cell lysates treated with anti-CLDN1 antibodies. (B) Western blot corresponding to membrane treated with anti-β-tubulin antibodies. [file Image1.PNG]
