# Supplementary material for: Disruption of Claudin-1 Expression by miRNA-182 Alters the Susceptibility to Viral Infectivity in HCV Cell Models
Source: Front Genet. 2018 Mar 20;9:93. doi: 10.3389/fgene.2018.00093 (PMC5869927; doi:10.3389/fgene.2018.00093)
Supplement: Supplementary file 2 [file DataSheet1.DOC]

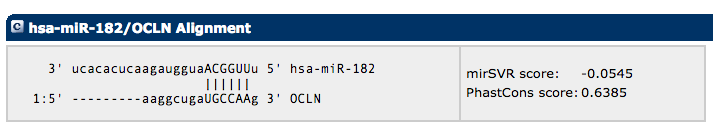


| **Genotype 4 targets** | **Gene** | **microRNA** | **Sites** | **Score** |
| --- | --- | --- | --- | --- |
| NS4B | gi|157781208:5413-6195 Hepatitis C virus genotype 4, genome | hsa-miR-182 | 1 | -3.76 |
| NS5A | gi|157781208:6196-7530 Hepatitis C virus genotype 4, genome | hsa-miR-182 | 3 | -9.9 |
| NS5B | gi|157781208:7531-9303 Hepatitis C virus genotype 4, genome | hsa-miR-182 | 2 | -0.011 |

| **Genotype 2 targets** | **Gene** | **microRNA** | **Sites** | **Score** |
| --- | --- | --- | --- | --- |
| NS2 | gi|157781212:2780-3430 Hepatitis C virus genotype 2, complete genome | hsa-miR-182 | 1 | -1.25 |
| NS4A | gi|157781212:5324-5485 Hepatitis C virus genotype 2, complete genome | hsa-miR-182 | 1 | 7.22 |
| NS4B | gi|157781212:5486-6268 Hepatitis C virus genotype 2, complete genome | hsa-miR-182 | 2 | -4.84 |
| NS5A | gi|157781212:6269-7666 Hepatitis C virus genotype 2, complete genome | hsa-miR-182 | 2 | -5.1 |
| NS5B | gi|157781212:7667-9439 Hepatitis C virus genotype 2, complete genome | hsa-miR-182 | 2 | -6.42 |
| C | gi|157781212:341-913 Hepatitis C virus genotype 2, complete genome | hsa-miR-182 | 1 | 3.04 |
